# Methylene Blue Infusion to Treat Severe Dapsone-Induced Methemoglobinemia in a Pediatric Patient

**DOI:** 10.7759/cureus.18853

**Published:** 2021-10-18

**Authors:** Bader Alyahya, Abdulaziz Alalshaikh, Ghofran Sabbahi, Mosaed Alnowiser, Mohammed Al-Mohawes

**Affiliations:** 1 Emergency Medicine, King Saud University, Riyadh, SAU; 2 Pediatric Infectious Diseases, King Abdulaziz University Hospital, Jeddah, SAU; 3 Emergency Medicine, King Saud University , Riyadh, SAU

**Keywords:** accidental ingestion, methylene blue infusion, toxicity, dapsone, methemoglobinemia

## Abstract

Dapsone overdose is a well-known potent cause of methemoglobinemia and hemolytic anemia. We discuss a case of a two-year-old male who developed severe persistent methemoglobinemia treated with multiple doses of methylene blue (MB), multidose activated charcoal, and vitamin C. Methylene blue infusion (rather than bolus dosing) aided in controlling this patient’s methemoglobin (MetHb) levels and symptoms and may reduce the total needed dose.

## Introduction

Dapsone is an antimicrobial agent commonly used to treat skin infections (such as leprosy) and dermatitis herpetiformis and as prophylaxis for *Pneumocystis jiroveci* pneumonitis [1,2]. Dapsone is often reported as a cause of acquired methemoglobinemia [3]. Other medications, such as benzocaine, sulfonamides, and nitrites, are also common inducers of methemoglobinemia [3]. Dapsone toxicity cases in children are usually a result of accidental ingestion. We describe a case of prolonged and recurrent methemoglobinemia despite treatment with multiple doses of methylene blue (MB), multidose activated charcoal, and vitamin C.

## Case presentation

A two-year-old male, weighing 13 kg, with no significant past medical history and no family history of glucose-6-phosphate dehydrogenase deficiency or hemoglobinopathy, arrived at the emergency department (ED) with his parents. He presented with irritability and bluish discoloration of his nails and lips. Initially, there was confusion about which medication the child ingested, as the parents kept the dapsone tablets in an omeprazole bottle. The father was using dapsone as a treatment for urticarial vasculitis. There was no history of co-ingestion in our patient, as the bottle contained no omeprazole tablets.

Due to the coronavirus disease 2019 pandemic, the patient’s parents were hesitant to go to a hospital and did not take him to the emergency department until eight hours after ingestion. Upon arrival, he had central and peripheral cyanosis, pallor, and irritability, and he was drowsy. His oxygen saturation was 60% on room air, heart rate was 170 beats/minute, respiratory rate was 40 breaths/minute, blood pressure was 117/64 mmHg, and random serum glucose was 5.3 mmol/L (95 mg/dL). The chest, cardiovascular, and abdominal examinations were unremarkable. 

He received 10 mL/kg of normal saline and was started on 15 L of oxygen via a non-rebreathing mask, which has improved his oxygen saturation to 75%. His venous blood gas showed a high methemoglobin (MetHb) level (48.4%), and his complete blood count demonstrated hypochromic microcytic anemia, as shown in Table 1. His liver and renal function test results were within reference ranges, and the findings from his chest X-ray were unremarkable.

**Table 1:** Initial laboratory results WBC, white blood cell; MCV, mean corpuscular volume; MCH, mean corpuscular hemoglobin; MCHC, mean corpuscular hemoglobin concentration; RDW, red cell distribution width

Analyte	Patient’s Result	Reference Range
Hemoglobin	8.8 g/dL	11–14 g/dL
WBC	6.20 x 10^9^/L	4–10 x 10^9^/L
Platelets	254.9 x 10^9^/L	150–400 x 10^9^/L
MCV	64 fL	81–97 fL
MCH	18.35 pg	27–31 pg
MCHC	320 g/L	320–360 g/L
RDW	14.08%	11.7%–14.8%

The regional poison control center was contacted, and based on their recommendation, as MB and the exchange transfusion experts were not immediately available, we administered a transfusion of packed red blood cells (PRBCs; 10 mL/kg) over three hours. The first dose of MB (1 mg/kg over five minutes) was given as soon as it became available (about three hours after his presentation). The patient’s peripheral cyanosis immediately improved after the first dose of MB, and his irritability resolved. One hour after his first dose of MB, the patient’s MetHb level decreased to 18%. He received two more boluses of MB (1 and 0.5 mg/kg) and was shifted to the pediatric intensive care unit. His MetHb levels and MB dosing are shown in Table 2. 

**Table 2:** Timeline of MetHb levels and MB doses MetHb, methemoglobin; MB, methylene blue

Day	Time	MetHb Level (%)	MB Dose
First day (May 21, 2021)	09:00	48.4%	Initial bolus (1 mg/kg)
	10:00	18%	1 mg/kg
	11:00	24%	0.5 mg/kg
	12:00	19.7%	0.5 mg/kg
	13:00	30%	1 mg/kg
	13:30	29.7%	0.5 mg/kg
	14:00	22%	0.5 mg/kg
	14:30	34%	MB infusion 2 mg/kg over 46.5 hours in fractionated periods (four hours of infusion and then observation of symptoms and MetHb level)
Second day	01:00	28.7%	
Third day	13:00	31.7%	MB infusion 1.5 mg/h for four hours
	21:00	27.7%	MB infusion 1.5 mg/h for four hours
Fourth day	07:00	31.1%	Exchange transfusion was initiated due to concerns of hemolysis
Fifth day	04:00	31.7%	MB infusion 1.5 mg/h over four hours
Sixth day	08:00	15.1%	Patient showed significant clinical improvement
Seventh day	04:00	11%	
Eighth day	08:00	2.5%	Patient was shifted to the pediatric ward for further observation

Unfortunately, the patient’s MetHb levels only temporarily improved after MB treatment; his MetHb levels relapsed after a few hours, so he received multiple doses of MB, multidose activated charcoal, multiple PRBC transfusions (a total of 30 mL/kg based on the recommendation of the hematologist), and ascorbic acid (100 mg orally, once daily). Despite several doses of MB and other treatments, his MetHb levels fluctuated and mainly were above 20%. Initially, the care team was targeting a MetHb level below 20% regardless of symptoms. After reaching a total dose of 5 mg/kg of MB, the aim of management shifted to minimizing the use of MB to avoid hemolytic anemia; therefore, a high level of MetHb was accepted (20%-30%) as long as the child was clinically well. We started MB infusion at 0.1 mg/kg/hour (rounded to 1.5 mg/hour) in fractionated periods, based on the MetHb level and the clinical manifestations. 

Three days following admission, the patient’s blood work showed some evidence suggestive of hemolysis (Table 3). Therefore, MB therapy was halted, and exchange transfusion was initiated as his MetHb level was still high (31.1%).

**Table 3:** Blood investigations for hemolysis LDH, lactate dehydrogenase; TBIL, total bilirubin; DBIL, direct bilirubin

Analyte	Patient’s Result	Reference Range
LDH	555 units/L	60– 170 units/L
TBIL	2.53 mg/dL	0.2–0.8 mg/dL
DBIL	0.75 mg/dL	0.02–0.75 mg/dL
Reticulocytes	1.3%	0.5%–1.5%
Absolute reticulocyte count	72 x 10^9^/L	0.25–0.75 x 10^9^/L

Six days post-ingestion, the patient started to show significant improvement in his clinical condition. Subsequently, he became asymptomatic, and his level of MetHb dropped to 15.1%. No additional doses of MB were required. After 48 hours of monitoring, the child remained stable; his oxygen saturation was 99% on room air, and his MetHb level was 2.5%. At this point, he was shifted to the pediatric medical ward for further observation, and two days later, he was discharged in stable condition.

## Discussion

Methemoglobinemia is a potentially fatal condition that arises from the oxidization of the ferrous iron (Fe2+) of heme to ferric iron (Fe3+). This results in an inability of oxygen to bind to hemoglobin, thus reducing oxygen-carrying potential in red blood cells, producing cyanosis and a characteristic chocolate brown venous blood. Furthermore, the unaffected hemoglobin binds avidly to oxygen, causing a decrease in oxygen release at the tissue level (producing a left shift of the oxygen-hemoglobin dissociation curve), contributing to tissue hypoxia [4].

Under normal circumstances, low MetHb levels of approximately 1% are maintained through regulatory mechanisms, including nicotinamide adenine dinucleotide-dependent cytochrome b-5 reductase, nicotinamide adenine dinucleotide phosphate (NADPH)-dependent methemoglobin reductase, reduced glutathione, and ascorbic acid. Cyanosis and the brown appearance of venous blood samples occur when levels of MetHb are approximately 10%-15% [5]. Further progress of cyanosis and the development of other symptoms, such as tachycardia, headache, fatigue, and weakness, occur with MetHb levels of 25%-50% [6]. MetHb levels higher than 70% are considered fatal [3,7]. Dapsone is known to cause methemoglobinemia and hemolytic anemia [1,8,9]. 

Management of methemoglobinemia can vary depending on the severity of the patient’s condition. Asymptomatic patients or patients with MetHb levels of less than 20% may only require simple measures such as stopping the causative agent. However, for symptomatic patients or patients with levels exceeding 20%, antidotal treatment with MB is indicated [10]. 

MB is deemed the primary treatment for significant methemoglobinemia due to dapsone toxicity; it acts as a cofactor in the NADPH system, which reduces MB to leucomethylene blue and subsequently reduces MetHb to hemoglobin [11,12]. MB is usually given intravenously as 1-2 mg/kg of a 1% solution over five minutes to treat methemoglobinemia. Additional doses of 1 mg/kg might be repeated for persistent cases [5]. However, exceeding a total dose of 7 mg/kg cumulatively is not recommended due to its potential to induce undesirable effects, such as hemolytic anemia and (paradoxically) methemoglobinemia [13,14]. In our patient, administering a small dose of MB as an infusion over four hours controlled his symptoms very well and helped reduce the total dose of MB. The patient received 5 mg/kg of MB as boluses in the first five hours and 3.4 mg/kg as an infusion over a total time of 113 hours in fractionated periods. Had we accepted higher levels of MetHb (as long as the patient was asymptomatic) and used infusion instead of boluses earlier, the total needed dose of MB would have been smaller. This finding is consistent with the finding of Berlin et al. in 1984 [15]. The safety and superiority of MB infusion over bolus dosing have been demonstrated by Dowson et al. in 1989 and Prasad et al.’s randomized controlled trial in 2008 [16,17]. 

In dapsone overdose, the elimination half-life can reach up to 80 hours, and it has significant enterohepatic recirculation. Therefore, multidose activated charcoal may enhance its elimination [12]. Previous reports have shown that repeated doses of activated charcoal have reduced dapsone mean half-life from 77 ± 23 to 12.7 ± 0.7 hours [8,12]. 

In our patient, reaching the diagnosis of methemoglobinemia was not a challenge in itself. Instead, the main challenges were identifying dapsone as the causing agent and dealing with its severe prolonged course, including multiple relapses. Although our patient received multiple doses of MB (for a total of 8.4 mg/kg) and multidose activated charcoal, he showed no signs of persistent recovery until the sixth day post-admission. 

## Conclusions

Dapsone toxicity is a very serious condition that requires prompt and effective management. Because of the long half-life of dapsone and its active metabolite, dapsone-induced methemoglobinemia can be severe and prolonged. MB infusion, rather than boluses, may be preferable in patients with dapsone-induced methemoglobinemia. 

## References

[1] Sangiolo D, Storer B, Nash R (2005). Toxicity and efficacy of daily dapsone as Pneumocystis jiroveci prophylaxis after hematopoietic stem cell transplantation: a case-control study. Biol Blood Marrow Transplant.

[2] Sago J, Hall III RP (2002). Dapsone. Dermatol Ther.

[3] Ash-Bernal R, Wise R, Wright SM (2004). Acquired methemoglobinemia: a retrospective series of 138 cases at 2 teaching hospitals. Medicine (Baltimore).

[4] Coleman MD, Coleman NA (1996). Drug-induced methaemoglobinaemia. treatment issues. Drug Saf.

[5] Ward KE, McCarthy MW (1998). Dapsone-induced methemoglobinemia. Ann Pharmacother.

[6] Barker SJ, Tremper KK, Hyatt J (1989). Effects of methemoglobinemia on pulse oximetry and mixed venous oximetry. Anesthesiology.

[7] Curry S (1982). Methemoglobinemia. Ann Emerg Med.

[8] Erstad BL (1992). Dapsone-induced methemoglobinemia and hemolytic anemia. Clin Pharm.

[9] Lee I, Barton TD, Goral S (2005). Complications related to dapsone use for Pneumocystis jirovecii pneumonia prophylaxis in solid organ transplant recipients. Am J Transplant.

[10] Chang YJ, Ali H, Draper A, Chua F (2013). An unusual cause of cyanosis in a patient with COPD. BMJ Case Rep.

[11] Canning J, Levine M (2011). Case files of the medical toxicology fellowship at Banner Good Samaritan Medical Center in Phoenix, AZ: methemoglobinemia following dapsone exposure. J Med Toxicol.

[12] Barclay JA, Ziemba SE, Ibrahim RB (2011). Dapsone-induced methemoglobinemia: a primer for clinicians. Ann Pharmacother.

[13] Fitzsimons MG, Gaudette RR, Hurford WE (2004). Critical rebound methemoglobinemia after methylene blue treatment: case report. Pharmacotherapy.

[14] Sunilkumar MN, Ajith TA, Parvathy VK (2015). Acute dapsone poisoning in a 3-year-old child: case report with review of literature. World J Clin Cases.

[15] Berlin G, Brodin B, Hilden JO, Mårtensson J (1984). Acute dapsone intoxication: a case treated with continuous infusion of methylene blue, forced diuresis and plasma exchange. J Toxicol Clin Toxicol.

[16] Dawson AH, Whyte IM (1989). Management of dapsone poisoning complicated by methaemoglobinaemia. Med Toxicol Adverse Drug Exp.

[17] Prasad R, Singh R, Mishra OP, Pandey M (2008). Dapsone induced methemoglobinemia : intermittent vs continuous intravenous methylene blue therapy. Indian J Pediatr.

